# Modified Smead–Jones suture for closure of emergency midline laparotomy incision: systematic review and meta-analysis

**DOI:** 10.1007/s13304-025-02192-3

**Published:** 2025-04-29

**Authors:** Mahmoud Diaa Hindawi, Ruaa Mustafa Qafesha, Ahmed Hamdy G. Ali, Hazim Alkousheh, Hatem Eldeeb, Haitham Salem, Abd-Elfattah Kalmoush, Amr Elrosasy

**Affiliations:** 1 Faculty of Medicine, Al-Azhar University, Cairo, Egypt; 2 Faculty of Medicine, Al-Quds University, Jerusalem, Palestine; 3 Faculty of Medicine, Ogarev Mordovia State University, Saransk, Russia; 4 Faculty of Medicine, Hashemite University, Zarqa, Jordan; 5 Faculty of Medicine, Ain Shams University, Cairo, Egypt; 6 General Surgery Department, Al-Azhar University, Cairo, Egypt; 7 Faculty of Medicine, Cairo University, Cairo, Egypt

**Keywords:** Laparotomy, Incisional hernia, Modified Smead Jones, Far-near-near-far, Wound dehiscence

## Abstract

Midline laparotomy incision is mostly used in emergent surgery, especially in hemodynamic instability patients. We aim to compare the Modified Smead–Jones (MSJ) and Smead–Jones (SJ) sutures against conventional continuous suture in midline laparotomy closure. PubMed, Scopus, Web of Science, and Ovoid were searched. We utilized Revman 5.4.1 for statistical analysis. Five studies involving 403 patients were included. Compared to continuous sutures, MSJ showed a significant reduction in wound dehiscence, wound infection, and hospital stay (RR = 0.29, 95% CI [0.14–0.59], p = 0.0006), (RR = 0.41, 95% CI [0.26–0.65], p = 0.0002), and (MD = – 4.50, 95% CI [– 5.43 to – 3.57], p = 0.00001). Conversely, the SJ subgroup showed no statistically significant difference in wound dehiscence, wound infection, and hospital stay. Also, both techniques, MSJ and SJ, showed no significant difference in incisional hernia risk (RR = 0.17, 95% CI [0.02–1.33], p = 0.09) and (RR = 5.16, 95% CI [0. 26–103.27], p = 0.28), respectively. MSJ follows the same far-near-near-far pattern as SJ but is applied continuously rather than interrupted. The MSJ suture technique might be promising in reducing wound dehiscence, infection, and hospital stay compared to conventional continuous closure. However, future large-scale RCTs with standardized methodologies and extended follow-up are essential to determine whether MSJ should be established as the preferred technique for midline laparotomy closure.

## Introduction

Midline laparotomy is widely used in both emergency and elective abdominal surgeries, serving as the primary approach for complex procedures due to its simplicity and the broad access it provides [1]. However, in emergency settings, particularly in patients with hemodynamic instability, the risks of incisional hernia (IH), postoperative abdominal wound dehiscence (WD) (burst abdomen), and surgical site infections are elevated, contributing to significant morbidity, impaired quality of life, and increased healthcare costs. These complications are influenced by multiple factors beyond abdominal wall closure, such as the patient's overall condition and the nature of the surgery [1, 2]. According to the recent guideline of the World Society of Emergency Surgery (WSES), there is a gap in this topic, and future studies are essential to clarify the optimal closure technique [3].

Recently, the evidence suggests that reinforced tension line (RTL) is the best technique as it decreases IH and AWD, and had the best SUCRA and p-score in the latest Network meta-analysis by Hernández et al. [4]. Currently, the Modified Smead–Jones suture (MSJ) is of great interest [5–9]. The MSJ is the (far-near-near-far) approach. Using a continuous suture rather than an interrupted one used in Smead–Jones suture (SJ), the rectus sheath, peritoneum, and muscle can be brought into approximation as a cohesive unit. The suture entered and left the body 2 cm from the borders of the wounds and 1 cm from either side of the edge of the linea alba [5].

A systematic review of the literature on MSJ and SJ is lacking. Here, we aim to be the first systematic review and meta-analysis to compare MSJ and SJ sutures' efficacy in comparison to continuous sutures by measuring the differences in wound infection, wound dehiscence, incisional hernia, and length of hospital stay. Providing valuable information for surgeons and researchers in this field.

## Methods

This systematic review and meta-analysis was registered in PROSPERO (CRD42024525979). We reported our work under the Preferred Reporting Items of Systematic Reviews and Meta-Analysis (PRISMA statement) guidelines [10]. We used the Cochrane Handbook of Systematic Reviews of Interventions as guidance [11].

### Eligibility criteria, search strategy, and data collection process

PubMed, Scopus, Web of Science, and Ovoid were searched (Appendix 1 in Supplementary information (SI)). We included Randomized controlled trials (RCTs) and observational studies (OS) that report emergency midline laparotomy incision patients closed with MSJ or SJ sutures in comparison to continuous suture. We excluded animal studies, theses, conference abstracts, and all single-arm studies. The MSJ and SJ techniques were performed according to their conventional definitions. The MSJ and SJ techniques were reported in the included studies according to their conventional definitions. The SJ technique uses interrupted sutures in the far-near-near-far pattern. The MSJ technique follows the same far-near-near-far pattern but in a continuous form. In all included studies, non-absorbable polypropylene sutures (Prolene 1 loop) were consistently used. The sutures were placed 2 cm from the wound edges and 1 cm from the linea alba [5–9]. The outcomes assessed were wound-related complications and hospitalization duration, systematically extracted from the included studies. Wound dehiscence was defined as the separation of the abdominal musculoaponeurotic layers within 30 days, requiring intervention. Wound infection referred to postoperative infection presenting with erythema, swelling, discharge, or requiring antibiotics. Incisional hernia was identified as a fascial defect at the laparotomy site, diagnosed clinically or radiologically. Hospital stay was measured as the number of days from surgery to discharge, reflecting recovery and postoperative complications [5–9].

### Quality assessment and statistical analysis

The risk of bias was assessed using the ROB-2 tool for the RCTs [12]. Newcastle–Ottawa Scale (NOS) quality assessments were used for OS [13]. The authors' independent blinded method was ensured in the whole study process: screening, data extraction, and quality assessment. We utilized RevMan 5.4.1 for statistical analysis. Pooled risk ratio (RR) and mean difference (MD) with 95% CI were used. Heterogeneity was assessed using I-square and *p*-value. The sensitivity analysis addressed heterogeneity.

### Reporting bias assessment

Publication bias assessment using the funnel plot method was not feasible, according to Egger el al. [14], there were not enough included studies as it requires a minimum of ten studies.

## Results

### Summary, baseline characteristics, and quality assessment of the included studies

After a comprehensive search of electronic databases, five studies were included [5–9], encompassing 403 patients with different hospital admission causes (Appendix 2 in SI). In all studies, non-absorbable sutures were used. The summary of these studies is represented in Table 1. Additionally, (Appendix 3 in SI) shows the detailed PRISMA flow diagram. The overall risk of bias was low as from three RCTs, all domains were assigned to be low risk of bias, and the two OS were of good quality (Appendix 4 in SI).

**Table 1:** Summary of the included studies

Study ID	Study design	Country	N	Closure technique study vs control	Suture type	Main inclusion criteria	Follow up	Conclusion
Garg (2023), [6]	Single-center prospective observational study	India	63	Smead-Jones technique vs. Continuous Closure	Polypropylene	Patients > 18 years undergoing emergency exploratory laparotomy procedures	21 months	The Smead-Jones technique for rectus sheath closure showed comparable outcomes to conventional closure methods. However, no significant differences were found in operative time, closure time, or postoperative complications
Aghara (2020), [7]	Single-center, prospective randomized clinical trial	India	100	Modified Smead-Jones technique vs. Continuous Closure	Polypropylene 1	Patients aged > 18 years who underwent emergency laparotomy through midline incision	12 months	The study showed that the Modified Smead Jones technique is better than the conventional continuous technique in midline laparotomy closure, regarding wound infection and dehiscence
Metawe (2023), [5]	Single-center, prospective randomized clinical trial	Egypt	50	Modified Smead-Jones technique vs. Continuous Closure	Polypropylene 1 number	Patients with risk factors for weak scar (being on hypoproteinemia, malignancy, malnutrition, immunosuppressant) and who underwent emergency laparotomy through midline incision	3 months	The modified Smead Jones technique performs better than the continuous technique when managing midline laparotomy closure in emergency laparotomy, especially regarding wound dehiscence and hospital stay
Nitin KB (2020), [8]	Single-center prospective case–control	India	90	Modified Smead-Jones technique vs. Continuous Closure	Synthetic, monofilament, non-absorbable polypropylene 1 number	Patients undergoing midline laparotomy between the age group of 18–70 years	15 days	The modified continuous Smead Jones technique of midline laparotomy wound closure has a low incidence of wound dehiscence and might also decrease the incidence of incisional hernia in the long term
Sringeri (2017), [9]	Single-blinded single-center randomized clinical trial	India	100	Modified Smead-Jones technique vs. Continuous Closure	Polypropylene number 1	Generalized peritonitis, irrespective of the cause and emergency laparotomy by midline incision in cases with delayed presentation (24 h after onset	6 months	The modified version of Smead-Jones techniques of laparotomy closure with propylene loop had a very low incidence of early and may reduce late complications. It was superior to other conventional methods of closure

Study characteristics, including study design, country, sample size (N), closure technique, suture type, main inclusion criteria, follow-up duration, and key conclusions

*N* number of patients

### Wound dehiscence

**Fig. 1:**
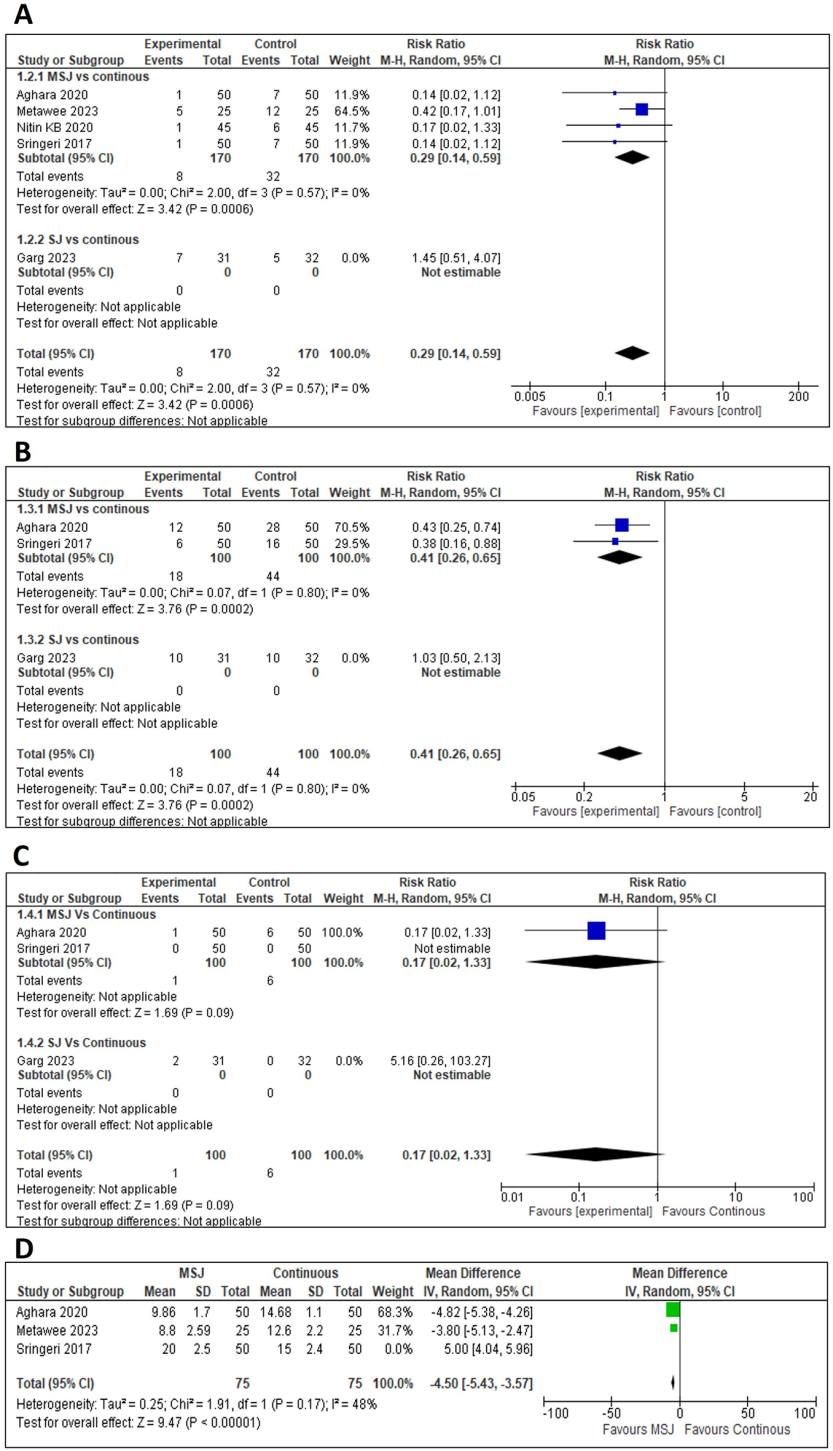
**Meta-analysis results of wound-related complications and hospital stay.** (A) **Wound dehiscence:** Risk ratio (RR) with 95% confidence interval (CI). (B) **Wound infection:** RR with 95% CI. (C) **Incisional hernia:** RR with 95% CI. (D) **Hospital stay:** Mean difference (MD) with 95% CI

In comparison to continuous sutures, MSJ showed a significant reduction in wound dehiscence (RR = 0.29, 95% CI [0.14–0.59], *p* = 0.0006). Conversely, the SJ subgroup showed no statistically significant difference, with increased risk in wound dehiscence (RR = 1.45, 95% CI [0.51–4.07], *p* = 0.49). The overall analysis, with a total of 403 patients, showed a significant reduction in both techniques (RR = 0.38, 95% CI [0.15–0.99], *p* = 0.05]. However, substantial heterogeneity was observed and resolved by the sensitivity analysis by excluding Garg et al. [6]. The results consistently supported the strength of the findings, demonstrating a significant decrease in the risk of wound dehiscence (Fig. 1A**).**

### Wound infection

MSJ showed a significant reduction in wound infection (RR = 0.41, 95% CI [0.26–0.65], *p* = 0.0002). Conversely, the SJ subgroup analysis showed the opposite (RR = 1.03, 95% CI [0.50–2.13], *p* = 0.93). The overall analysis showed a significant reduction in wound infections (RR = 0.55, 95% CI [0.30–1.01], *p* = 0.05), with moderate heterogeneity. A sensitivity analysis was conducted by excluding Garg et al. [6]. The results consistently supported the strength of the findings, demonstrating a significant decrease in the risk of wound infection (Fig. 1B).

### Incisional hernia

Both subgroups, MSJ and SJ, showed no significant difference in incisional hernia risk (RR = 0.17, 95% CI [0.02–1.33], *p* = 0.09) and (RR = 5.16, 95% CI [0.26–103.27], *p* = 0.28), respectively. Similarly, the overall analysis did not show any significant difference (RR = 0.78, 95% CI [0.03–22.12], *p* = 0.88). There was substantial heterogeneity, and sensitivity analysis was conducted by excluding Garg et al. [6], which maintained non-significant results (Fig. 1C).

### Hospital stay

The analysis showed no significant difference in hospital stay between MSJ and continuous (MD = – 1.21, 95% CI [– 7.67, 5.26], *p* = 0.71). Considerable heterogeneity was observed, and sensitivity analysis was conducted by excluding Sringeri et al. [9], demonstrating a significant reduction in hospital stay with MSJ.

## Discussions

MSJ follows the same far-near-near-far pattern as SJ but is applied continuously rather than interrupted [5–9]. To our knowledge, this is the first systematic review and meta-analysis that reports MSJ and SJ against conventional continuous suture. MSJ results demonstrated promising significant efficacy in reducing wound dehiscence, infection and limiting hospital stay time. Also, although not statistically, comparable results were found in reducing incisional hernia. Heterogeneity in some outcomes required sensitivity analyses. The ideal closure method maintains the tensile strength through the healing process [2]. The process of wound healing can be categorized into three distinct phases [2]. The initial exudative phase (days 1–4) does not contribute to the wound's tensile strength. Following that, the proliferative phase (days 5–20) follows, during which the tissue regenerates around 15–30% while up to 80% of its original tensile strength is restored in the subsequent remodeling phase (day 21 onwards) [2]. This explains our results as the MSJ technique uses a 'far-near-near-far' approach, which enhances the approximation of the rectus sheath, peritoneum, and muscle layers [5]. Also ensures that the suture grabs a larger amount of tissue away from the incision line (far) before coming closer (near), which enhances the wound’s mechanical stability and reduces the tension on the wound edges, thus lowering the risk of wound dehiscence [15, 16]. Also, by securing the suture farther from the incision line, there is less stress on the wound edges during movement, which is particularly important in the abdominal area where tension is naturally higher due to respiratory movements and intra-abdominal pressure. Reduction in wound dehiscence and infection rates directly correlates with shorter hospital stays. Fewer complications mean less need for prolonged medical intervention or additional surgical procedures, thus allowing for quicker patient discharge. However, IH is influenced by factors such as the patient’s age, nutritional status, and overall tissue integrity rather than just the suture technique [17]. The strength of the closure in the long-term, particularly past the remodeling phase of healing, is contingent on factors like collagen synthesis and tissue remodeling, which are not solely influenced by suture technique [2]. An observational study conducted by Garg et al. (2023) explained the variability in wound dehiscence, infection, and incisional hernia, attributing the effects to surgeon-determined closure techniques, baseline comorbidity differences, and shorter follow-up. Sringeri et al. (2017) introduced hospital stay heterogeneity through the generalized peritonitis population with delayed presentation, which led to longer hospitalization in the MSJ group. Such exclusions highlight the importance of such markers for future randomized controlled trials, which have to be better comparable.

### Strengths, limitations and implications

This is the first systematic review and meta-analysis addressing MSJ as a promising suture technique. We made a comprehensive literature review highlighting evidence in this topic as well as limitations for future research to work on. This study points out that MSJ might potentially reduce wound dehiscence, infection, and hospital stays. However, the small sample size (five studies with 403 patients) limits the statistical power of this meta-analysis, while the restricted number of studies prevented a formal publication bias assessment, as funnel plot analysis and Egger’s test require a minimum of ten studies. Heterogeneity was observed, driven by differences in study design, follow-up duration, and baseline comorbidity variations, which may have influenced outcomes. The absence of long-term data prevented conclusions regarding hernia recurrence because of the significant variation in follow-up periods. Important factors for muscle closure, such as tension on the fascia, the ratio of short-length suture to long-length suture, muscle approximation, peritoneal handling, and surgeon expertise, were inconsistently reported and potentially introduced additional sources of variability. To solve this, future research should aim for large-scale multicenter RCTs with standardized inclusion criteria to minimize heterogeneity.

Long-term follow-up studies are essential to assess hernia recurrence and provide comprehensive data on postoperative complications. Furthermore, detailed reporting of surgical techniques, including fascial tension control, suture bite size and distance, muscle approximation, and peritoneal handling, will improve the comparability of studies. Conducting biomechanical studies to test for tensile strength and closure integrity will reinforce the evidence base. Well-designed studies with consistent methodologies and longer follow-up durations will ultimately be needed to ascertain whether MSJ should be promoted as the preferred closure technique for emergency midline laparotomy.

## Conclusion

The MSJ suture technique might be promising in reducing wound dehiscence, infection, and hospital stay compared to conventional continuous closure. However, future large-scale RCTs with standardized methodologies and extended follow-up are essential to determine whether MSJ should be established as the preferred technique for midline laparotomy closure.

## Data Availability

All data relevant to this study are included in this article.
